# Graph theory analysis of whole brain functional connectivity to assess disturbances associated with suicide attempts in bipolar disorder

**DOI:** 10.1038/s41398-021-01767-z

**Published:** 2022-01-10

**Authors:** Anjali Sankar, Dustin Scheinost, Danielle A. Goldman, Rebecca Drachman, Lejla Colic, Luca M. Villa, Jihoon A. Kim, Yarani Gonzalez, Imani Marcelo, Mei Shinomiya, Brian Pittman, Cheryl M. Lacadie, Maria A. Oquendo, R. Todd Constable, Hilary P. Blumberg

**Affiliations:** 1 Department of Psychiatry, Yale School of Medicine, New Haven, CT USA; 2 Department of Radiology and Biomedical Imaging, Yale School of Medicine, New Haven, CT USA; 3 Interdepartmental Neuroscience Program, Yale School of Medicine, New Haven, CT USA; 4 Department of Psychiatry and Psychotherapy, Jena University Hospital, Jena, Germany; 5 Department of Psychiatry, University of Oxford, Oxford, UK; 6 Department of Psychiatry, Perelman School of Medicine, University of Pennsylvania, Philadelphia, PA USA; 7 Child Study Center, Yale School of Medicine, New Haven, CT USA

**Keywords:** Bipolar disorder, Human behaviour

## Abstract

Brain targets to lower the high risk of suicide in Bipolar Disorder (BD) are needed. Neuroimaging studies employing analyses dependent on regional assumptions could miss hubs of dysfunction critical to the pathophysiology of suicide behaviors and their prevention. This study applied intrinsic connectivity distribution (ICD), a whole brain graph‐theoretical approach, to identify hubs of functional connectivity (FC) disturbances associated with suicide attempts in BD. ICD, from functional magnetic resonance imaging data acquired while performing a task involving implicit emotion regulation processes important in BD and suicide behaviors, was compared across 40 adults with BD with prior suicide attempts (SAs), 49 with BD with no prior attempts (NSAs) and 51 healthy volunteers (HVs). Areas of significant group differences were used as seeds to identify regional FC differences and explore associations with suicide risk-related measures. ICD was significantly lower in SAs than in NSAs and HVs in bilateral ventromedial prefrontal cortex (vmPFC) and right anterior insula (RaIns). Seed connectivity revealed altered FC from vmPFC to bilateral anteromedial orbitofrontal cortex, left ventrolateral PFC (vlPFC) and cerebellum, and from RaIns to right vlPFC and temporopolar cortices. VmPFC and RaIns ICD were negatively associated with suicidal ideation severity, and vmPFC ICD with hopelessness and attempt lethality severity. The findings suggest that SAs with BD have vmPFC and RaIns hubs of dysfunction associated with altered FC to other ventral frontal, temporopolar and cerebellar cortices, and with suicidal ideation, hopelessness, and attempt lethality. These hubs may be targets for novel therapeutics to reduce suicide risk in BD.

## Introduction

Suicides in the United States are increasing at an alarming rate. Bipolar disorder (BD) is the disorder that carries the highest suicide risk. Approximately 50% of persons with BD make a suicide attempt [1], and 15–20% die by suicide [2], a rate that is 20–30 times higher than that of the general population [3]. Elucidation of the neuropathophysiology of suicide behavior in BD and brain targets for more effective prevention strategies are urgently needed.

Neuroimaging studies have begun to probe the brain circuitry underlying suicide thoughts and behaviors (STBs), and the most convergent findings have been in the ventromedial prefrontal cortex (vmPFC), a region that subserves emotion regulation [4]. In particular, lower function in the vmPFC has previously been shown to be associated with emotion regulation difficulties [5] and STBs in BD [2, 6]. Findings in vmPFC connection sites have also been reported, including other ventral PFC (vPFC) regions, dorsal PFC, insula, mesial temporal lobe and striatum, implicating abnormalities in a distributed vmPFC system subserving emotion regulation in both BD and STBs [2, 6–8]. Emerging evidence suggests that these may be transdiagnostic features for STBs, as abnormalities in emotion regulation and vmPFC system have shown associations with STBs in major depressive disorder and schizophrenia [2, 9].

There are few functional neuroimaging studies of STBs in BD. Only a subset implemented functional connectivity (FC) approaches [6, 10–12] and these predominantly relied on a priori assumptions about involved brain regions [6, 10, 12]. In this nascent field, it is important not to have premature closure on involved brain regions which could lead to missing important hubs of dysfunction. Moreover, most studies that investigated suicide risk for their associations with brain function have focused on constructs of behavioral dyscontrol, such as impulsivity [13]. While impulsivity can lower the threshold to suicide behaviors, its study may not reflect subjective experiences such as hopelessness and ideation that drive suicide behavior.

**Fig. 1:**
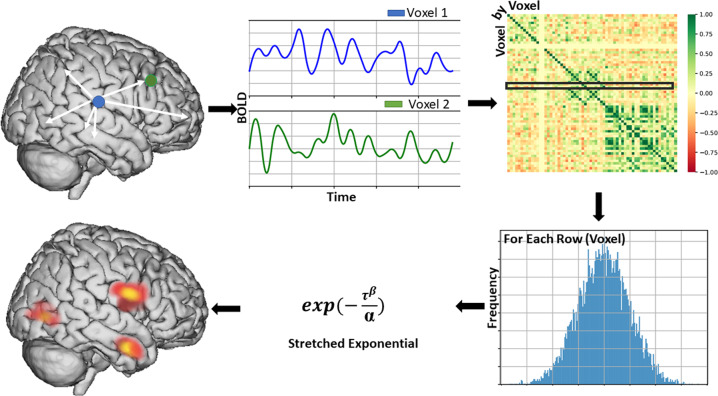
Overview of the intrinsic connectivity distribution analysis method. Intrinsic Functional Connectivity of each voxel, as measured by the Intrinsic Connectivity Distribution (ICD), is calculated by correlating the time series for a grey matter voxel with every other voxel’s time course. This procedure is repeated for every voxel, resulting in a voxel-by-voxel correlation matrix. From this matrix, a single row is extracted, which represents all correlations to a voxel, and converted to a histogram to estimate the distribution of connectivity for that voxel. From this distribution, a survival function is constructed and fitted with a stretch exponential with unknown parameters α and β. For our purposes, α controls the rate of decay of the survival function with a larger α indicating a slower decay and larger global connectivity. The α ICD maps can then be thresholded resulting in a whole-brain parametric image that reveals putative hubs of the individual connectome.

Here, we used a whole-brain voxel-wise graph‐theoretical measure, the intrinsic connectivity distribution (ICD [14]) (Fig. 1), to map functional disturbances associated with suicide attempts in BD and explore their associations with subjective suicide risk-related domains. This novel method has been used in the study of FC disturbances in major depressive disorder [15, 16]. ICD provides a measure of FC of each voxel to every other voxel in the brain and does not rely on a priori seed information. Furthermore, it does not use an arbitrary threshold to define a connection. Instead, correlation thresholds are estimated using a Weibull distribution generating parametric images of whole-brain FC measures. ICD allows examination of the distribution of connections to a voxel instead of investigating only a few strong connections as in other graph theory-based measures [14]. This method has shown improved sensitivity to detect group differences over previous graph theory-based methods [14] as well as improved sensitivity to detect subtle differences in FC between participants which may be lost with other data-driven methods that were used to study STBs in mood disorders [17] such as Independent Component Analysis [18]. While most graph-theory methods to date have been performed on resting-state data, ICD has been used successfully to identify behaviorally-relevant functional disturbances using salient fMRI task data [19, 20]. Salient fMRI tasks have been shown to reveal disorder-relevant differences between participants in patterns of FC more so than resting state [21]. Given the evidence for maladaptive emotion regulation processes and disruptions in the underlying vmPFC emotion regulation brain circuitry in BD [22], an emotional face gender-labeling task shown to engage the vmPFC circuitry [6] was utilized in this study to identify brain functional disturbances associated with suicide behavior in BD. This fMRI task has also shown to elicit differences in functional responses and connectivity in the vmPFC circuitry in studies of BD, [6, 23, 24] including in studies with suicide attempters. [6] Since the participants with BD had made a suicide attempt at least two months prior to their neuroimaging scan, the study aimed to capture neurofunctional features that are associated with a history of suicide behavior.

To our knowledge, this is the first study to use ICD in adults with BD to map the whole-brain functional disturbances associated with suicide behavior while individuals performed a task involving implicit emotion regulation processes important in both BD and STBs. As the most consistent findings for STBs have been in the vmPFC [2], and as ICD is based on a whole brain graph-theoretical approach, we hypothesized that participants with BD and prior history of suicide attempts (SAs) would show altered ICD in regions including, but not limited to, vmPFC, relative to BD participants with no prior attempts (NSAs) and healthy volunteers (HVs). We anticipated ICD disturbances would be associated with subjective suicide risk-related domains, namely suicidal ideation, and hopelessness severity.

## Materials and methods

### Participants

Participants for this study were recruited through advertisements placed in the community, and through referrals from surrounding in-patient and out-patient programs. Eighty-nine participants met criteria for BD according to the Diagnostic and Statistical Manual of Mental Disorders (Fourth ed. Text Revision; DSM-IV-TR) [ages 18–55 years, mean age ± standard deviation (SD) = 29.2 ± 10.7 years; 67.4% female]. The SA group (*n* = 40; 26.7 ± 8.5 years; 77.5% female) was comprised of BD participants who were assessed using the Columbia Suicide History Form [25] and met criteria for an “actual” attempt in which the self-injurious act was carried out with some intent to die regardless of medical lethality.

In the SA group, 45% (*n* = 18) of the subjects reported one past suicide attempt, while the others (55%, *n* = 22) reported multiple attempts (range = 2 to >10 attempts); 23% (*n* = 9) of the SA group had an attempt within the past one year. The NSA group (*n* = 49; 30.9 ± 12.0 years; 59.2% female) was comprised of BD participants who denied a history of an actual suicide attempt. The presence of Axis I diagnoses and mood state in BDs were confirmed with the Structured Clinical Interview for DSM-IV Diagnosis (SCID) [26]. The HV group [*n* = 51; ages 18–55 years, mean age ± SD = 34.5 ± 12.4 years; 60.8% female] was comprised of participants without a lifetime history of Axis-I disorders confirmed with the SCID, without first degree relatives with major mood, substance/alcohol abuse or dependence or psychotic disorders, assessed using the Family History Screen for Epidemiological Studies [27], and without lifetime history of STBs assessed using the SCID and the Columbia Suicide History Form. Exclusion criteria for all participants were major medical disorders (except treated hypothyroidism in 1 SA and 2 NSA participants), central nervous system conditions including a history of loss of consciousness ≥5 min, or substance or alcohol abuse or dependence within three months. Written informed consent after receiving a complete description of the study was obtained from participants in accordance with the Yale School of Medicine Human Investigation Committee institutional review board. Table 1 summarizes demographic and clinical information.

**Table 1:** Participant demographic and clinical features.

	Subjects with bipolar disorder				Healthy volunteers		Significance
	BD-SA (*N* = 40)		BD-NSA (*N* = 49)		HV (*N* = 51)		
	Mean	SD	Mean	SD	Mean	SD	*p*
Age (years)	26.7^*^	8.5	30.9	12.0	34.5	12.4	0.005
HDRS-29	11.6^*^	9.9	10.5^◊^	8.9	0.5	1.1	<0.001
YMRS	5.6^*^	5.6	5.5^◊^	5.9	0.3	0.6	<0.001
Hopelessness^a^	7.8^*^	5.2	6.4^◊^	5.3	1.9	1.9	<0.001
Suicidal ideation^a^ (most severe)	19.3^+^	8.2	5.9	7.6			<0.001
Suicide intent (most recent attempt)	11.1	6.3					
Suicide intent (most lethal attempt)	12.0	5.5					
Highest lethality^a^	2.46	2.1					
	**Range**						
Time since most recent attempt	2mo–18yr						
Time since most lethal attempt	2mo–18yr						
	***N***	**%**	***N***	**%**	***N***	**%**	***P***
Females	31	77.5	29.0	59.2	31	60.8	0.14
Handedness:							0.46
Right	36.0	90.0	39.0	79.6	46.0	90.2	
Left	3.0	7.5	9.0	18.4	4.0	7.8	
Ambidextrous	1.0	2.5	1.0	2.0	1.0	2.0	
Mood state at scan:							0.80
Euthymic	15.0	37.5	21.0	42.9			
Depressed	11.0	27.5	14.0	28.6			
Elevated	14.0	35.0	14.0	28.6			
BDI (vs BDII)	38.0^+^	95.0	38.0	77.6			0.02
Rapid cycling^a^	17.0	43.6	20.0	41.7			0.86
Current psychosis	3.0	7.5	2.0	4.1			0.49
Lifetime psychosis	14.0	35.0	18.0	36.7			0.87
Unmedicated at scan	16.0	40.0	23.0	46.9			0.51
Medications							
Anticonvulsants	12.0	30.0	14.0	28.6			0.88
Antipsychotics	7.0	17.5	16.0	32.7			0.10
Antidepressants	9.0	22.5	9.0	18.4			0.63
Benzodiazepines	5.0	12.5	9.0	18.4			0.45
Stimulants	6.0	15.0	3.0	6.1			0.17
Lithium carbonate	5.0	12.5	7.0	14.3			0.81
Adrenergic agonists	1.0	2.5	0	0.0			0.27
Non-benzodiazepine hypnotic	2.0	5.0	2.0	4.1			0.84
Levothyroxine	1.0	2.5	2.0	4.1			0.86
*Comorbidity*							
Lifetime substance use disorders	23.0	57.5	22.0	44.9			0.24
Alcohol dependence	10.0	25.0	6.0	12.2			0.12
Alcohol abuse	3.0	7.5	7.0	14.3			0.31
Cannabis dependence	7.0	17.5	4.0	8.2			0.18
Cannabis abuse	5.0	12.5	5.0	10.2			0.73
Cocaine dependence	4.0	10.0	6.0	12.2			0.74
Cocaine abuse	1.0	2.5	1.0	2.0			0.88
Opiate dependence	1.0	2.5	1.0	2.0			0.88
Polysubstance dependence	2.0	5.0	2.0	4.1			0.84
Lifetime other psychiatric disorders							
Post-traumatic stress disorder	12.0	30.0	8.0	16.3			0.12
Panic disorder	7.0	17.5	5.0	10.2			0.32
Social phobia	4.0	10.0	5.0	10.2			0.98
Generalized anxiety disorder	3.0	7.5	6.0	12.2			0.46
Specific phobia	6.0	15.0	2.0	4.1			0.07
Obsessive-compulsive disorder	1.0	2.5	6.0	12.2			0.09
Anorexia nervosa	2.0	5.0	3.0	6.1			0.82
Eating disorders NOS	1.0	2.5	2.0	4.1			0.68
Binge eating disorder	2.0	5.0	0	0.0			0.11
Bulimia nervosa	0	0.0	1.0	2.0			0.36

*BD-SA* Participants with Bipolar Disorder (BD) who met criteria for at least one “actual” suicide attempt, *BD-NSA* Participants with BD who had no history of actual suicide attempts, *HV* Healthy Volunteers, *HDRS-29* 29-item Hamilton Depression Rating Scale, *YMRS* Young Mania Rating Scale, *NOS* Not Otherwise Specified, *SD* Standard Deviation, *N* Number of cases, *%* Percentage of cases.

No subject met criteria for substance/alcohol abuse or dependence within three months.

*p < 0.05 for SA vs. HV, ^+^*p* < 0.05 for SA vs. NSA, ^◊^*p* < 0.05 for NSA vs. HV, ^a^Scores not available: BHS for 3 SA, 5 NSA, 1 HV; suicide ideation for 5 SA, 2 NSA; lethality for 1 SA; rapid cycling for 1 SA, 1 NSA; *mo* months, yr years.

Mood symptom severity was assessed using the Hamilton Depression Rating Scale 29-item version (HDRS-29) [28] and the Young Mania Rating Scale (YMRS) [29]. Hopelessness severity was assessed with the Beck Hopelessness Scale (BHS) across all participants [30], and most severe suicidal ideation severity with the Beck Scale for Suicidal Ideation (SSI) in the BD group [31]. Participants in HV group were also administered the SSI and all participants had a score of zero indicating that were without lifetime history of suicidal ideation. For SAs, lethality of the most lethal attempt was assessed with the Beck Medical Lethality Scale [32], and intent to die of both the most recent and most lethal lifetime attempt with the Beck Suicide Intent Scale [32].

### MRI data acquisition

A 3T Trio was used for MR scanning (Siemens, Erlangen, Germany). A high-resolution 3-dimensional scan was obtained using a magnetization-prepared rapid gradient-echo (MPRAGE) sequence (repetition time (TR): 1500 ms; echo time (TE): 2.77 ms; flip angle (FA): 15°; matrix: 256 × 256; field of view (FOV): 256 × 256 mm^2^; slice thickness, 1.0 mm without gap; 160 contiguous slices) and non-linearly registered to the template brain. Two-dimensional T1-weighted images were also obtained (TR: 300 ms; TE: 2.47 ms; FA: 60°; matrix: 256 × 256; FOV: 256 × 256 mm^2^; slice thickness: 3 mm without gap) and used for linear registration to the MPRAGE image. Functional data were collected with a T2*-weighted single-shot echo planar imaging sequence with Blood Oxygen Level-Dependent contrast, aligned with the anterior commissure-posterior commissure plane (TR: 2000ms; TE: 25 ms; FA: 80°; matrix: 64 × 64; FOV: 240 × 240 mm^2^; slice thickness: 3.0 mm without gap; 32 contiguous slices) and linearly registered to the two-dimensional anatomical image.

FMRI data were obtained while participants performed an event-related emotional face gender-labeling task in which subjects viewed male or female faces from the Ekman series depicting happy, fearful, or neutral expressions and pressed a button to indicate a male-female determination. Five male and 5 female faces were each presented for 2 seconds (sec) with interstimulus intervals of 4, 8, or 12 sec. In each run lasting 4 min and 50 sec, each of the three expressions was shown for each actor, for a total of 30 face stimuli. Subjects performed four consecutive runs of the task. The order of face stimuli was varied systematically to control for sequential dependencies and runs were counterbalanced for the expression, gender, and identity of the faces, as well as for the interstimulus intervals. [6, 33]

### Intrinsic connectivity distribution processing

The first four volumes of each functional run were discarded to allow for hemodynamic steady state. Motion correction was performed; all participants had an average framewise displacement of < 0.2 mm. Images were warped into common space using non-linear transformation and iteratively smoothed at 6 mm full-width at half-maximum using AFNI’s 3dBlurToFWHM (afni.nimh.nih.gov/afni/) [34]. Further preprocessing steps were performed using BioImage Suite (bioimagesuite.org). Covariates of no interest regressed from the data included linear and quadratic drifts, mean cerebrospinal fluid, white matter, and mean gray matter signals, and 24-parameter motion variables (including six rigid-body motions parameters, six temporal derivatives, and their squares). Connectivity was calculated based on the “raw” task time courses, i.e., without regressing out the task-evoked activity. The data were temporally smoothed with a Gaussian filter (~cutoff frequency = 0.12 Hz). A grey matter mask in Montreal Neurological Institute (MNI) space was applied so only voxels in the grey matter were used for further calculations [35]. The four task-based time courses were variance normalized and concatenated and the ICD of each voxel was calculated for each subject [35].

### Intrinsic connectivity distribution estimation

After preprocessing, intrinsic functional connectivity of each voxel, as measured by the ICD [14], was calculated by correlating the time series for each grey matter voxel with the time series of every other grey matter voxel in the brain. A summary statistic based on network theory measure ‘degree’ was calculated. Degree is a common network theory measure used to analyze fMRI data such as resting-state that characterizes the number of connections between a given ROI or voxel and every other ROI or voxel [36]. Instead of simply integrating the strength of all connections for a particular voxel, as is performed in the degree metrics, ICD models the distribution of the connection strength. Thus, ICD represents a generalization of the degree metric incorporating information about the survival of the distribution of the connection strength instead of just the area under the curve [14]. ICD avoids the need for choosing arbitrary connectivity as it models the distribution of correlation thresholds using a Weibull distribution: $\frac{\beta }{\alpha }\left( {\frac{r}{\alpha }} \right)^{\beta - 1}exp\left( { - \left( {\frac{r}{\alpha }} \right)^\beta } \right)$, where *r* is a correlation between the two-time series, is the variance parameter, and is the shape parameter. This parameterization is akin to modeling change in network theory metric *degree*, as the threshold utilized to calculate *degree* is increased, with a stretched exponential: ${{{\mathrm{exp}}}}\left( { - \frac{{\tau ^\beta }}{\alpha }} \right)$, where *τ* is the correlation threshold, and *α*, *β* are the parameters as above.

A histogram of these correlations was constructed to estimate the distribution of connections to each voxel, which were converted to survival functions. Each point on the survival function is simply degree, based on a binary graph. The survival function was estimated as $1 - {{{\textit{F}}}}\left({{{\textit{x}}}}\right)$, where ${{{\textit{F}}}}\left({{{\textit{x}}}}\right)$ is the cumulative sum of the histogram, and was fitted with a stretched exponential with unknown variance. As variance controls the spread of the distribution of connections, a larger variance indicates a greater number of high correlation connections. This process was repeated for all voxels in the grey matter resulting in a whole-brain parametric image for each participant summarizing the connectivity of each tissue element.

### Seed-based functional connectivity processing

Seed-to-whole brain FC analyses were performed to explore regional connectivity patterns contributing to between-group ICD differences. The seed regions were defined using a 10 mm cube around significant clusters from the ICD analysis [19, 37]. For each participant, the time course for each voxel was computed separately for each seed. These time courses were correlated with the time course for every other voxel in the gray matter to create a map of *r*-values, which were transformed to *z*-values using Fisher’s transform, yielding one map for each participant for each seed that represented the strength of its correlations to other regions.

### Data analyses

#### Demographic and clinical measure analyses

Analyses were conducted using the Statistical Package for the Social Sciences 26.0 (SPSS-26) to examine if there were potential differences between the three groups in any of the demographic or clinical measures. Potential group differences in continuous (age) and categorical (gender, handedness) demographic measures were assessed using analysis of variance (ANOVA) with post hoc two-tailed independent-samples t-tests, and chi-square tests, respectively. Potential group differences in non-normally distributed continuous (HDRS-29, YMRS, BHS) clinical measures present in SA, NSA, and HV groups were assessed using Kruskal-Wallis tests with *post hoc* two-tailed Mann-Whitney *U*-tests. Potential differences between SA and NSA groups in continuous (SSI) and categorical [mood state, rapid cycling, current and history of psychosis, BD subtype (I/II), current medications overall and subtypes, comorbidities) were analyzed using non-parametric two-tailed Mann-Whitney *U-*tests and chi-square tests, respectively (except for variables with at least one expected count of <5, for which a Fisher’s exact test was used instead) to examine if the two BD groups differed on clinical measures other than on STB measures.

#### FMRI analyses

##### Between-group analyses

Analysis of covariance (ANCOVA) was performed in Statistical Parametric Mapping version 12 (SPM12) to examine differences in ICD with group (SA, NSA, and HV) as the between-subjects factor, and age as a covariate owing to the wide age range of the participants and significant group difference in age and associated brain changes. Analogous models were used for seed-based analyses. Results for both ICD and seed-based analyses were considered significant if they survived a cluster-based correction for multiple comparisons of *p* < 0.05 [initial voxel-level threshold *p* < 0.001, family-wise error (FWE)-corrected; *p*_*fwe-cluster*_ < 0.05]. For clusters showing significant differences among the three groups (main effect of group) in both the ICD and seed-based analyses, mean ICD and seed-based connectivity values were extracted for each individual using MarsBaR to perform *post hoc* between-group comparisons using two-tailed independent samples *t* (for normally distributed ICD and seed-based connectivity values) and Mann-Whitney *U* (for non-normally distributed ICD and seed-based connectivity values) tests. Results were Bonferroni-corrected for the 3 pairwise group comparisons.

#### Exploratory analyses of relationships between demographic and clinical factors and ICD

Connectivity values from clusters that showed significant group differences in the ICD analysis were also explored for potential effects of gender in all participants using two-tailed independent samples *t* or Mann-Whitney *U* tests. In BD participants, potential effects of clinical factors were explored using independent samples *t* or Mann-Whitney *U* tests when there were at least 10 participants with and 10 participants without the clinical factor by adding each clinical factor one at a time as a between-subject factor. Clinical factors explored in this study were mood state (euthymic/symptomatic), BD subtype (I/II), rapid cycling, lifetime psychosis, medications overall and subclasses (anticonvulsants, antipsychotics, antidepressants, benzodiazepines, lithium), past substance use disorders (alcohol dependence, alcohol abuse, cannabis dependence, cannabis abuse, cocaine dependence), and comorbidity (post-traumatic stress disorder, panic disorder). In the SA group, potential effects of the number of suicide attempts (one *vs*. multiple attempts) on connectivity values were explored using independent samples *t* or Mann-Whitney *U* tests.

Exploratory correlational analyses were performed between connectivity values from each cluster that showed significant group differences in the ICD analysis and suicide risk-related measures (BHS, SSI, suicide intent of most recent and lethal attempt, and highest attempt lethality). Analyses were conducted across all participants groups for BHS, across BD participants for SSI, and in the SA group for intent and attempt lethality, using Spearman (r_s_) or Kendall’s tau (r_τ_) correlations as appropriate. Exploratory analyses above were considered significant at *p* < 0.05, uncorrected.

## Results

### Demographic and clinical measures

Groups differed significantly in age (F(2, 137) = 5.42, *p* = 0.005); the HV group was significantly older than the SA group (t(89) = 3.4, Bonferroni corrected *p*_*corr*_ < 0.004). The groups did not significantly differ in gender (*p* = 0.14) or handedness (*p* = 0.46). Groups significantly differed in HDRS-29 (H(2) = 73.6, *p* < 0.001), YMRS (H(2) = 45.6, *p* < 0.001), and BHS (H(2) = 39.0, *p* < 0.001) scores. The HV group had significantly lower HDRS-29, YMRS and BHS than SA (HDRS: U = 158.5, *p*_*corr*_ < 0.001; YMRS: U = 347, *p*_*corr*_ < 0.001; BHS: U = 246.5, *p*_*corr*_ < 0.001) and NSA (HDRS: U = 187, *p*_*corr*_ < 0.001; YMRS: U = 428, *p*_*corr*_ < 0.001; BHS: U = 501, *p*_*corr*_ < 0.001) groups. BHS scores did not differ significantly between SA and the NSA groups, but the SA group had significantly higher SSI scores than the NSA group (U = 211.5, *p* < 0.001). The SA group had a greater proportion of BDI individuals compared with the NSA group (χ_1_^2^ = 5.38, *p* = 0.03). There were no significant differences between the SA and NSA groups on any of the other clinical factors that were explored (*p* > 0.1).

### FMRI analysis

#### ICD

**Fig. 2:**
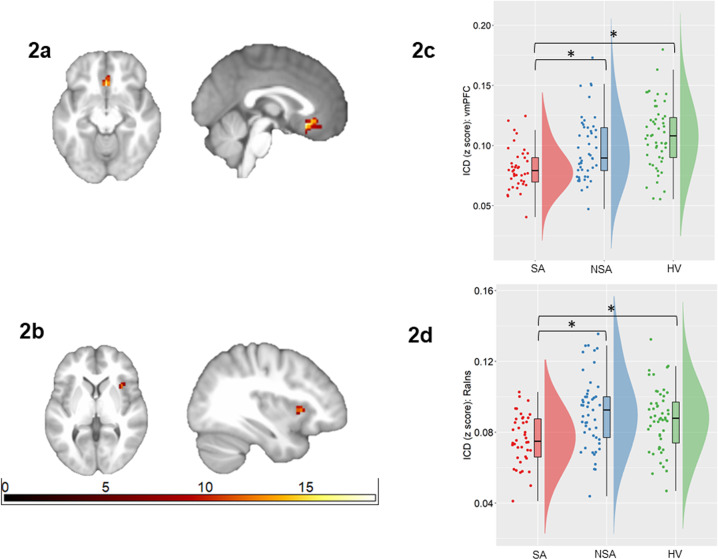
Alterations in intrinsic connectivity distribution associated with suicide attempts in bipolar disorder. The axial and sagittal images show regions of differences in intrinsic connectivity distribution from an ANCOVA analysis comparing individuals with bipolar disorder with history of suicide attempts (SA group; *n* = 40), individuals with bipolar disorder without suicide attempts (NSA group; *n* = 49), and healthy volunteers (HV; *n* = 51), in bilateral ventromedial prefrontal cortex (vmPFC; Fig. 2a) and right anterior insula (RaIns; Fig. 2b) (*p* < 0.05, corrected for family-wise error). Left of the figure denotes left side of brain. The color bar represents the range of *F* values. The raincloud plot [63] of distribution of extracted z-values from the vmPFC (Fig. 2c) and the RaIns (Fig. 2d) show the group differences resulted from lower ICD in these regions in the SA group compared to the NSA and HV groups (*p < 0.05, corrected for 3 pair-wise group comparisons).

ICD in the bilateral caudal vmPFC (Brodmann Areas (BAs) 11, 25) and right anterior insula (RaIns) differed among SA, NSA, and HV groups. In particular, ICD in vmPFC was significantly lower in the SA than the NSA (U = 611, *p*_*corr*_ = 0.009) and HV (U = 413, *p*_*corr*_ < 0.001) groups. Intermediate values were observed in the NSA group that did not significantly differ from the HV group (*p*_*corr*_ = 0.11). ICD in the RaIns region was significantly lower in the SA group than the NSA (*t*(87) = −3.99, *p*_*corr*_ < 0.001) and HV (*t*(89) = −3.25, *p*_*corr*_ = 0.006) groups, while no significant difference was observed between NSA and HV groups (*p*_*corr*_ = 0.72). Figure 2a–d.

#### Effects of demographic and clinical factors on ICD

There were no significant effects of gender (*p* ≥ 0.34). Participants with a history of alcohol dependence had significantly lower ICD in RaIns than those without (*p* = 0.02). There were no significant effects of other clinical factors including mood state, BD subtype, rapid cycling, psychosis, medications overall and subclasses (anticonvulsants, antipsychotics, antidepressants, benzodiazepines, lithium), alcohol abuse, cannabis dependence, cannabis abuse, cocaine dependence, and comorbidity. In the SA group, there was no significant effect of number of attempts on ICD.

Across all participants, BHS scores were associated with bilateral vmPFC ICD (r_s_ = −0.23, *p* = 0.008). Within BD, SSI scores were negatively associated with bilateral vmPFC (r_s_ = −0.23, *p* = 0.040) and RaIns (r_s_ = −0.27, *p* = 0.012) ICD. Within SAs, highest attempt lethality was negatively associated with bilateral vmPFC ICD (r_τ_ = −0.25, *p* = 0.039) (Supplementary Fig. S1). There were no significant effects of other suicide risk-related measures including suicide intent of the most recent or most lethal attempt, or number of suicide attempts on ICD (*p* ≥ 0.20)

#### Seed connectivity

**Fig. 3:**
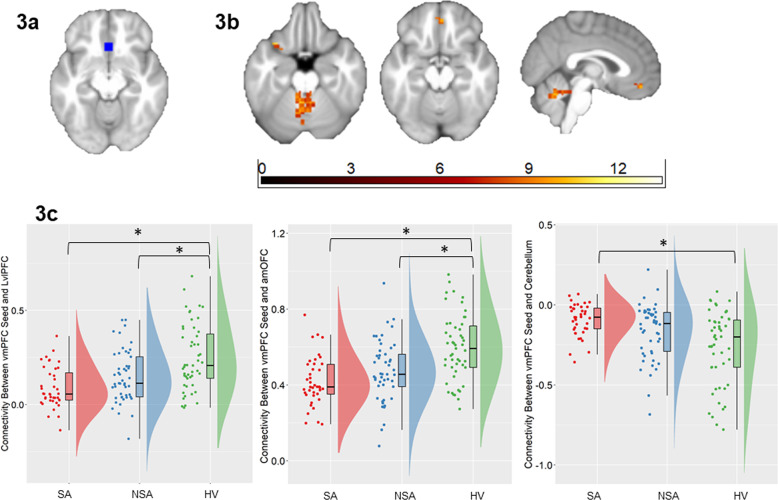
Alterations in functional connectivity from the ventromedial prefrontal cortex associated with suicide attempts in bipolar disorder. The axial and sagittal images show regions of differences in functional connectivity from the ventromedial prefrontal cortex (vmPFC) seed region (Fig. 3a) to the left ventrolateral prefrontal cortex (LvlPFC), anteromedial orbitofrontal cortex (amOFC), and cerebellar vermis (Fig. 3b), using an ANCOVA analysis comparing individuals with bipolar disorder with history of suicide attempts (SA group; *n* = 40), individuals with bipolar disorder without suicide attempts (NSA group; *n* = 49) and healthy volunteers (HV; *n* = 51) (*p* < 0.05, corrected for family-wise error). Left of figure denotes left side of brain. The color bar represents the range of *F* values. The raincloud plots (Fig. 3c) of distribution of extracted z-values from vmPFC-LvlPFC and vmPFC-amOFC clusters of differences in functional connectivity show the group differences resulted from lower connectivity from vmPFC to LvlPFC and to amOFC in the SA and the NSA groups, compared to the HV group. The raincloud plot (Fig. 3c) of distribution of extracted z-values from vmPFC-cerebellum show the group differences resulted from lower negative functional connectivity in the SA group compared to the HV group (*p* < 0.05, corrected for 3 pair-wise group comparisons).

**Fig. 4:**
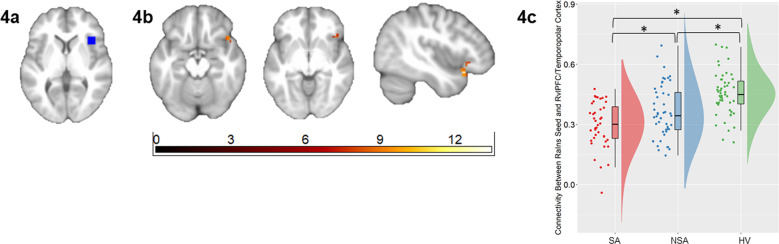
Alterations in functional connectivity from the right anterior insula associated with suicide attempts in bipolar disorder. The axial and sagittal images show regions of differences in functional connectivity from the right anterior insula (RaIns, a region of the olfactocentric paralimbic cortex (OPC)) seed region (Fig. 4a) to the right ventrolateral prefrontal cortex (RvlPFC) and right temporopolar regions of the OPC (Fig. 4b) using an ANCOVA analysis comparing individuals with bipolar disorder with history of suicide attempts (SA group; *n* = 40), individuals with bipolar disorder without suicide attempts (NSA group; *n* = 49) and healthy volunteers (HV; *n* = 51) (*p* < 0.05, corrected for family-wise error). Left of figure denotes left side of brain. The color bar represents the range of *F* values. The raincloud plot (Fig. 4c) of distribution of extracted z-values from the RaIns-RvlPFC/temporopolar cluster show the group differences resulted from lower connectivity in the SA group compared to the NSA and HV groups (*p* < 0.05, corrected for 3 pair-wise group comparisons).

Bilateral vmPFC (Fig. 3a) and RaIns (Fig. 4a) regions were used as seeds for follow-up FC analyses. The SA, NSA, and HV groups differed in their connectivity from the vmPFC to the left ventrolateral prefrontal cortex (LvlPFC, BA47), bilateral anteromedial orbitofrontal cortex (amOFC, BA11) and bilateral cerebellum particularly in the vermis (Fig. 3b). In particular, compared with the HV group, SA and NSA groups both had lower vmPFC-LvlPFC (U ≥ 470, *p*_*corr*_ ≤ 0.016) and vmPFC-amOFC (U ≥ 446, *p*_*corr*_ ≤ 0.001) FC. The magnitude of FC to both these regions were intermediate for the NSA group, but did not differ significantly between SA and NSA groups (*p*_*corr*_ ≥ .20) (Fig. 3c). Compared with the HV group, the SA group had reduced vmPFC-cerebellum negative connectivity (U = 550, *p*_*corr*_ < 0.001). The magnitude of FC to the cerebellum was intermediate for the NSA group, though did not significantly differ from the SA or HV groups (*p*_*corr*_ ≥ 0.14) (Fig. 3c).

Connectivity from the RaIns to a cluster encompassing RvlPFC (BA47) and right temporopolar cortices (BA38) (Fig. 4b) also differed among SA, NSA, and HV groups. In particular, the SA group had lower RaIns-RvlPFC/temporal pole FC relative to both NSA (t(87) = −2.50, *p*_*corr*_ = 0.042) and HV (t(89) = −6.59, *p*_*corr*_ < 0.001) groups. Intermediate values were observed in the NSA group that differed significantly from the HV group (t(98) = −3.68, *p*_*corr*_ = 0.001) (Fig. 4c).

## Discussion

A graph‐theoretical measure, ICD that models the distribution of all connections to a voxel, was used to examine FC disturbances in SAs, compared to NSAs and HVs, while they performed a task involving implicit emotion regulation processes important in both BD and STBs. SAs showed significantly reduced ICD (i.e., reduced whole brain connectivity) in bilateral vmPFC and RaIns relative to NSAs and HVs. Reduced ICD in vmPFC and RaIns was associated with suicidal ideation, and reduced ICD in vmPFC was additionally associated with hopelessness and attempt lethality. Seed-based connectivity analyses from the vmPFC and RaIns regions of ICD differences were performed to identify major projection sites to which their FC were altered. In these seed-based connectivity analyses, the vmPFC showed significantly reduced FC with the vPFC (amOFC and LvlPFC) and reduced negative FC with the cerebellar vermis in SAs relative to HVs. FC of RaIns with the RvlPFC and right temporopolar cortices was also significantly reduced in SAs relative to NSAs and HVs. These findings implicate the vmPFC and RaIns as central hubs of dysfunction within brain systems of dysfunction that include additional PFC, temporal, and cerebellar regions, in STBs of BD.

VmPFC (BAs 11, 25), a site of reduced ICD, along with amOFC and vlPFC, sites of its FC decreases, are important nodes of the system posited to subserve healthy emotion regulation [38]. Dysfunction within these vPFC regions has been previously observed in individuals with BD in response to emotion regulation tasks [5] and is thought to reflect maladaptive emotional regulation, a core feature of BD implicated in generating its high rate of STBs [39]. Functional and structural imaging studies have shown vPFC alterations associated with suicide attempts in adults and adolescents with mood disorders [6, 40]. The vmPFC was one of the most discriminating regions identified in a multivariate pattern recognition analysis of responses to concepts related to death and life, that distinguished adolescent ideators who made a past suicide attempt from those who did not [41]. In the present study, vmPFC ICD values were intermediate in the NSA group, suggesting vmPFC may be a hub of dysfunction contributing to vulnerability to STBs in BD.

The findings of this study suggest that in individuals with BD, vmPFC dysfunction may relate not only to suicide behavior but also to subjective feelings and thoughts that may drive potentially lethal suicide behavior. Reduced vmPFC ICD was associated with hopelessness, suicidal ideation, and attempt lethality. Hopelessness is a relatively understudied construct, although it is a major suicide risk factor that predicts suicidal ideation [42] and is associated with attempt lethality [43]. The ICD findings expand our recently reported finding of a relationship between impaired vmPFC functioning during emotion regulation and hopelessness in BD [5]. We speculate that a hub of vmPFC dysfunction may impair emotion regulation processes and hinder the ability to inhibit negative self-focused and pessimistic thinking, leading to increases in hopelessness which in turn puts individuals at heightened risk for suicidal ideation and lethal suicidal behavior.

There is now ample data that subregions of the cerebellum, including the vermis, are involved in the experience and regulation of emotions [44, 45] through its reciprocal connections with limbic and prefrontal regions. The literature also suggests it is involved in a bias towards processing more negative than positive emotional stimuli [46]. In the SA group, we found reduced negative connectivity between the vmPFC and cerebellum. This reduced anticorrelation between the vmPFC and cerebellar nodes might suggest a reduced ability to switch from the intense processing of emotions to their regulation in response to negative emotional stimuli.

The ICD approach also revealed the RaIns as a potential novel hub of dysfunction associated with suicide behavior and suicidal ideation. Functional neuroimaging studies have seldom focused on the aIns as a region of interest in the study of BD or STBs, although altered FC to the insula from other seed regions (e.g. limbic or PFC) has been reported in association with suicide behavior in mood disorders [11, 47, 48]. These findings suggest that investigating dysfunctions in the aIns is important to fully understand the neuropathophysiology of STBs. The aIns, especially the right side, is implicated in the experience of salient emotions that derive from information about internal bodily states [49]. Perception of physical and psychological pain have been shown to alter activity in the aIns [50, 51], and activate the mu-opioid receptor system in the aIns [50, 52]. Moreover, higher levels of psychological pain are also associated with STBs [53]. Thus, RaIns hub of dysfunction could reflect altered perception of salient painful and other interceptive experiences that could lead to the generation of STBs. Whether these dysfunctions are due to dysregulation of the endogenous opioid system is unknown and would need to be confirmed in future studies. If confirmed, it could suggest a possible neurotransmitter target to prevent suicide. Potential further ties between the RaIns finding and the endogenous opioid system are their links to substance use disorders including alcohol dependence [54, 55]. In this study, ICD in RaIns was lower in individuals with a history of alcohol dependence compared to those without. However, as the sample size of individuals with a history of alcohol dependence in this study was limited (*n* = 16), this finding must be considered with caution.

The vPFC, insula, and temporopolar regions comprise the olfactocentric paralimbic cortex (OPC) [56, 57], a group of highly interconnected brain regions that integrate internal and external information for adaptive emotion regulation. We previously observed OPC structural abnormalities in adolescents with BD [58]. As OPC regions share developmental features [57], we suggested that OPC might be important in the neurodevelopment of BD. In a previous study of STBs in BD [6], using seed-based analyses from the amygdala, we observed vPFC FC disturbances but did not detect insula and temporopolar FC abnormalities as in the current whole-brain approach. The current clustering of findings in the OPC using the ICD approach provides new support for the potential importance of the OPC in the neurodevelopment of STBs.

The findings from the present study should be considered in the context of its limitations. This study has a cross-sectional design; therefore the association of findings to future risk cannot be concluded. However, one of the only longitudinal studies examining future suicide risk in BD, albeit in a modest sample, found vPFC alterations in future attempters compared to non-attempters, suggesting that vPFC alterations may be indicative of future risk [59]. Further study is also required to better understand the brain functional correlates associated with the transition from ideation to behavior. Only 23% of the individuals in the SA group were recent attempters, defined by the presence of an actual attempt in the past year, which made it difficult to perform meaningful analyses to examine whether recency of an attempt had an impact on ICD findings. The present study was performed only in individuals with BD which limits the generalizability of findings to the wider population of suicide attempters; however, there have been reports of these regions being associated with STBs in major depression and schizophrenia as well [2]. Moreover, paths to suicide behavior may vary by individual characteristics. Although previous studies have showed potential effects of medications such as stimulants and antipsychotics on brain FC [60–62], we did not detect a significant effect. The sample size in the present study may have limited power to detect potential effects of medications. Exploration of medication effects on the brain findings was further limited as participants were often on more than one psychotropic medication, and the medications were not systematically controlled. Apart from attempt history, the SA and the NSA groups differed on the severity of suicidal ideation, and on the proportion of BDI to BD II individuals. As expected, the SA group had higher ratings on the SSI than the NSA group, and had higher proportion of individuals who were BDI relative to the NSA group. Post-hoc analyses did not reveal significant effects of clinical factors on ICD findings; however, the sample size in the present study may have limited power to detect potential effects of the clinical factors studied. Longitudinal multivariate pattern recognition studies with large samples sizes and that examine whether vmPFC and aIns hubs of dysconnectivity can predict suicide behavior at the individual level are needed.

In conclusion, this study used a whole-brain graph-theoretical approach to investigate FC disturbances in suicide attempters with BD and explore their associations with STBs. Findings point to the vmPFC and RaIns as being hubs of dysfunction with altered connections to other ventral frontal, temporopolar, and cerebellar cortices. FC differences in the vmPFC observed even in the absence of a priori seeds and network definitions lend further support to the mounting evidence that this region may have a key role in the pathophysiology of suicide behavior. The approach also provides new leads for the field as it provides novel evidence for the aIns as a hub of dysfunction important in the neuropathophysiology of STBs in BD. The study, thus, provides new evidence for the vmPFC and aIns as targets for novel therapeutics to reduce the high levels of hopelessness, suicide ideation, and suicide behavior, and thereby the high risk of suicide in individuals with BD.

## Supplementary information

Supplemental Material
