# Supplementary material for: Graph theory analysis of whole brain functional connectivity to assess disturbances associated with suicide attempts in bipolar disorder
Source: Transl Psychiatry. 2022 Jan 10;12:7. doi: 10.1038/s41398-021-01767-z (PMC8748935; doi:10.1038/s41398-021-01767-z)
Supplement: Supplementary file 1 — Supplemental Material [file 41398_2021_1767_MOESM1_ESM.docx]

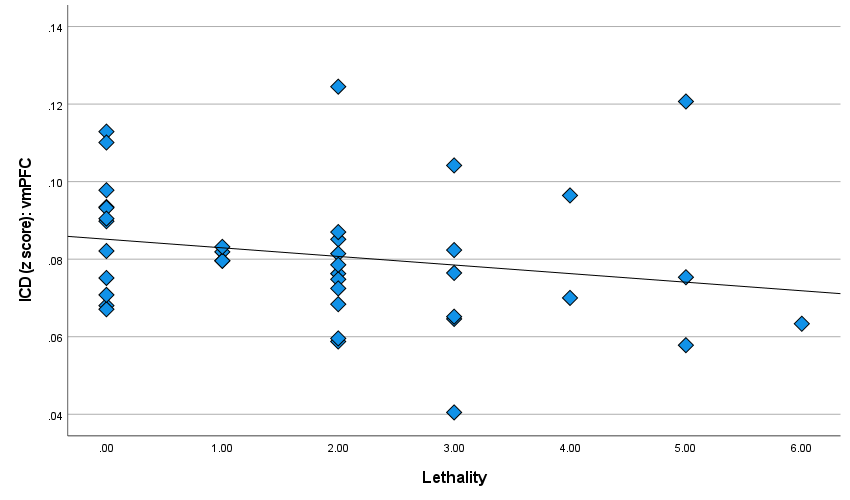
**Supplementary Figure S1**

**Figure S1: The Association Between Ventromedial Prefrontal Cortex Functional Connectivity and Severity of Lethality**

The graph shows significant negative associations between ventromedial prefrontal cortex (vmPFC) functional connectivity and severity of lethality (r_τ_=-.25, *p*=.039). The *y* axis presents the z-values extracted from the vmPFC cluster which showed lower ICD in the SA group compared to the NSA and HV groups, and the *x* axis presents lethality of the most lethal suicidal attempt scores in the SA group. Raw values are presented for variables in the *x* and *y* axis, and the goodness-of-fit line is for illustrative purposes only.
